# From knowledge as domination to knowledge as governmentality: a theoretical rearticulation of power in late-modern knowledge regimes

**DOI:** 10.3389/fsoc.2026.1799909

**Published:** 2026-04-24

**Authors:** Marcos Parada-Ulloa, Jorge Bozo Marambio, Hugo Carmona, Óscar Vega Gutiérrez

**Affiliations:** 1 Departamento de Educación, IICSE, Universidad de Atacama, Copiapó, Chile; 2 Facultad de Salud y Ciencias Sociales, Universidad de las Américas, Santiago, Chile; 3 Departamento de Educación, Universidad de Atacama, Copiapó, Chile; 4 Departamento de Trabajo Social, Universidad Tecnológica Metropolitana, Santiago, Chile

**Keywords:** governance, knowledge–power, late modernity, legitimation, normalization

## Abstract

The article addresses a central problem in contemporary sociology: the inadequacy of classical approaches to power in explaining how expert knowledge currently organizes forms of domination in societies marked by the expansion of expertocracy, neoliberal rationality, and algorithmic mediation. Although traditions such as critical theory, the sociology of power, and Foucauldian genealogy have analyzed the relationship between knowledge and domination, a conceptual gap persists regarding the mechanisms through which knowledge simultaneously operates as legitimation, normalization, and a technology of governance. The aim of the study is to conceptually rearticulate the nexus of knowledge–power in order to explain how expert knowledge structures authority, organizes conduct, and administers populations in late modernity. The theoretical framework integrates contributions from Weber, Bourdieu, Gramsci, and critical theory with the Foucauldian perspective on power–knowledge and governmentality, while also incorporating insights from the sociology of scientific knowledge and Science and Technology Studies (STS), particularly in relation to expertise, quantification, and sociotechnical infrastructures. Methodologically, the research was conducted as a documentary study with a theoretical–conceptual orientation, based on a genealogical analysis of foundational and contemporary texts. The procedure involved comparative reading, critical problematization, and typological reconstruction of the mechanisms through which knowledge produces effects of power. The results identify three interdependent mechanisms that articulate the power of knowledge: legitimation, through the production of epistemic authority; normalization, through the establishment of metrics, standards, and classifications; and governance, through the integration of these infrastructures into population management devices based on data and algorithms.

## 1 Introduction

The political, social, and economic transformations of the twenty-first century have substantively reconfigured the relationship between knowledge and power. In the classical formulation associated with Francis Bacon (1,620/2000), knowledge was conceived as a lever to expand human intervention over nature, sustaining the modern promise of progress and rational control. In late modernity, however, knowledge does not merely explain or represent the social world: it organizes it. It functions as an infrastructure of legitimacy, normalization, and governance, producing categories, defining thresholds of acceptability, and shaping conduct through institutional, expert, and technical devices that permeate the state, the market, and everyday life (Foucault, 1975/1995; Weber, 1922/2021).

In this context, the maxim “knowledge is power” ceases to be a general philosophical statement and becomes a sociological and political problem. Knowledge intervenes in the distribution of resources, the hierarchization of subjects and territories, and the stabilization of inequalities through cultural repertoires that present themselves as neutral—evidence, efficiency, quality, or risk—but operate as criteria of classification and authority. Understanding how power manifests through knowledge requires analyzing the social mechanisms by which certain forms of knowledge acquire institutional legitimacy, orient decisions, and produce effects of normalization in everyday life (Kolakowski, 1985).

Sociology possesses a robust tradition for thinking about power. Weber (1922/2021) demonstrated that administrative rationalization produces forms of legitimate domination based on legal-rational authority, where technical knowledge becomes a central resource of governance. Critical theory emphasized how instrumental reason can operate as a device of domination when technical criteria are presented as inevitable (Horkheimer and Adorno, 2002). Bourdieu (2001), in turn, analyzed knowledge as cultural and symbolic capital capable of classifying, hierarchizing, and distributing legitimacy across social fields. In his approach, knowledge not only classifies positions abstractly but acts through institutionalized practices—such as academic accreditation, professional certification, or evaluation systems—that transform cognitive competencies into recognized authority and establish hierarchies of competence in professional and administrative domains.

A similar logic is observed in the concentration of expert power analyzed by Mills (1956/2005). His notion of the power elite helps explain how certain actors concentrate organizational, political, and cognitive resources that grant them the capacity to influence strategic decisions. In the contemporary context, this process is expressed in the centrality of experts, technocrats, and specialized agencies that define political agendas based on technical diagnoses. The relevant mechanism here is not only the concentration of power but the ability to convert specialized knowledge into a legitimate criterion for public decision-making.

Gramsci’s (1975) notion of hegemony complements this analysis by showing how expert authority acquires social acceptance. Knowledge becomes effective when it succeeds in presenting itself as universal, neutral, or technically necessary. This process of naturalization constitutes a mechanism of consent production: political decisions appear as inevitable technical solutions rather than contestable normative choices. Hegemony, therefore, also operates at the epistemic level, transforming cognitive frameworks into shared horizons of meaning.

The sociology of scientific knowledge has deepened this perspective by showing that expert authority depends not only on formal credentials but also on social practices of validation. Harry Collins (1985, 2010) demonstrated that the production and circulation of scientific knowledge largely depend on forms of tacit knowledge that cannot be fully formalized. Expertise is thus socially constituted through situated learning processes, communities of practice, and shared recognition criteria. Collins and Evans (2007) further distinguish different degrees of expertise, allowing us to understand how certain actors acquire authority to intervene in technical debates, particularly in public policy formulation.

Michel Foucault (1969/1979, 1975/1995) provides an indispensable analytical key by conceiving power as practice. His notion of governmentality shows how knowledge operates through devices that produce subjects and orient conduct. Knowledge functions as a technology of conduct, articulating statistics, expert diagnoses, and administrative procedures that enable intervention upon populations. Power does not merely repress: it produces subjectivities, delimits what can be said, and defines regimes of truth.

Under current conditions of cognitive capitalism, expertocracy, and algorithmic mediation, governance mechanisms based on knowledge are intensified through systems of classification, scoring, prediction, and uncertainty management. Indicators, audits, rankings, and evaluation models do not merely describe social reality: they establish behavioral standards and orient institutional decisions. This entails recognizing a mutation in the operation of power. Without abandoning economic domination or institutional coercion, a regime of governance through knowledge expands, acting upon populations and subjects via technical criteria and institutional narratives that produce reasonable obedience and practical consent. The central problem lies in understanding how the technical acquires moral and political status, and how governing through instruments of knowledge becomes legitimate when these appear to stand beyond conflict.

Despite extensive theorization on the knowledge-power nexus, contemporary sociology still lacks an integrated conceptual framework to explain how expert knowledge simultaneously operates as domination, normalization, and governance in societies shaped by expertocracy, cognitive capitalism, and algorithmic mediation. This gap becomes visible in phenomena such as governance by indicators, technocratic risk management, the datafication of social life, and the automation of public and private decisions—dynamics that exceed explanations focused exclusively on coercion, ideology, or class.

In this context, the present article seeks to intervene in this conceptual gap by clarifying how knowledge becomes a technology of governance that produces subjectivities, organizes hierarchies, and stabilizes inequalities through culturally legitimized instruments. The research question is oriented toward explaining which mechanisms allow expert knowledge to function simultaneously as a source of legitimacy, a matrix of normalization, and a technology of governance in everyday life and in the institutions of late modernity. Building on this, the article proposes a conceptual rearticulation of the knowledge–power nexus that integrates the sociocritical tradition and the genealogical approach to explain how expert knowledge operates as legitimation, normalization, and governance in contemporary societies.

## 2 Methodology

### 2.1 Approach and design

This research is documentary in nature, employing a conceptual-genealogical analysis design. Its objective is not to produce primary data, but rather to reconstruct and theoretically rearticulate the nexus between expert knowledge and power. The genealogical approach, inspired by Foucault, entails tracing the provenance and emergence of concepts, attending to their conditions of possibility, their ruptures, and their recompositions across different problem fields, rather than seeking an essence or a linear origin.

### 2.2 Systematic delimitation of the corpus

To ensure the traceability of the analysis, a textual corpus was constructed and organized into three concentric layers, selected according to criteria of theoretical relevance and influence within the contemporary debate on knowledge-power.

The selection is not exhaustive but rather strategic. Peripheral debates were deliberately excluded in order to maintain a clear focus on the axis of knowledge–power–government.

### 2.3 Analytical procedure and conceptual extraction matrix

The analysis unfolded in three iterative phases, employing a conceptual extraction matrix (Andréu, 2002) as a systematization tool.

Phase 1: Open coding of foundational texts (Layers 1 and 2). A line-by-line reading of the classical and developmental texts was conducted. The objective was to identify and extract all explicit or implicit propositions regarding how knowledge is linked to power. The extraction matrix was operationalized through the following guiding questions for each text:

- What definition or conceptualization of power is proposed?
- What status and function are assigned to knowledge (ideology, capital, technology, truth)?
- Through which specific mechanisms does this relationship operate? (e.g., in Weber, bureaucracy and technical knowledge; in Foucault, examination and surveillance; in Bourdieu, symbolic violence and classification).
- In which institutional devices is it materialized? (e.g., school, prison, census, credential).

Phase 2: Selective coding and Constant comparison (Layers 2 and 3). Building on the initial codes (legitimacy, classification, discipline, etc.), a directed reading of contemporary sociological texts (Layer 3) was undertaken. The purpose was to trace how classical mechanisms were transformed, reappeared, or displaced in the analysis of phenomena such as quantification, auditing, or expertise (Collins and Evans, 2007). The constant comparison technique was employed: each new emergent code (e.g., “indicator reactivity,” “tacit knowledge”) was contrasted with codes from previous phases to refine definitions and avoid overlaps.

Phase 3: Synthesis and typological reconstruction.

The constant comparison across the three corpus layers revealed a recurrent pattern: the operation of power–knowledge could be grouped into three distinct yet interdependent analytical dimensions. Thus, based on the coded textual evidence, a typological model was reconstructed to structure the results of this article:

- Legitimation: Codes such as “epistemic authority,” “technical neutrality,” “credentialism” (derived from Weber, Bourdieu, Collins).
- Normalization: Codes such as “standards,” “metrics,” “classification,” “production of the normal” (derived from Foucault, Bowker and Star, Power).
- Governance: Codes such as “population management,” “risk,” “data infrastructures,” “algorithmic decision-making” (derived from Foucault/Rose, Porter, and analyses of the literature on datafication).

Validation of the analysis.

The validity of the proposed model does not rest on its correspondence with an externally measurable reality, but rather on its internal coherence (the logical consistency of the relationships among categories), its heuristic fruitfulness (its capacity to shed light on diverse phenomena such as governance through indicators in health, education, or security), and its traceability (the possibility for another researcher to follow the path from texts to categories, as explicitly outlined in this procedure) (Fernández, 2002).

## 3 Knowledge as an articulator of power in global and complex societies

In the context of a globalized and complex society, knowledge emerges as a central axis in the articulation and legitimation of power. It is not merely that knowledge accompanies power as an external justification; rather, it operates as social infrastructure: it produces authority, defines criteria of truth, establishes standards of conduct, and enables forms of indirect governance. The issue is not whether knowledge influences power, but which mechanisms allow expert knowledge to function simultaneously as legitimation, normalization, and a technology of governance in late modernity.

From the classical tradition, Maquiavelo (1532/2010) and Hobbes (2003) demonstrate that political power requires not only force but also the capacity to order and anticipate conduct through principles of state rationality. Locke (1689/2006), in turn, opens the problem of legitimacy as normative agreement. Yet in modern society, legitimacy no longer rests exclusively on the social contract or sovereignty, but on the authority of specialized knowledges that claim neutrality. With Weber (1922/2021), this mutation becomes evident: legal-rational domination relies on bureaucracies whose foundation is technical competence. Expert knowledge legitimizes authority by transforming political decisions into “technical” decisions, displacing conflict into a terrain administered by credentials, protocols, and reports. Thus, knowledge configures the contemporary form of obedience, presented as reasonable because it is anchored in notions of “evidence,” “efficiency,” or “quality.”

If Weber (1922/2021) explains the link between rationalization and authority, Bourdieu specifies the mechanism by which knowledge becomes socially effective power: by functioning as cultural and symbolic capital, it classifies, hierarchizes, and distributes legitimacy, defining what counts as competence, merit, or excellence (Bourdieu, 1998/2001). Knowledge organizes the social world through categories that become naturalized as universal. Symbolic violence, in this sense, is not an accidental excess but a structural effect of the imposition of legitimate meanings: the “correct,” the “competent,” the “normal.” Institutions such as schools, universities, or professional systems transform evaluation into a technology of classification: they do not merely measure, but produce differentiated subjects, possible trajectories, and boundaries of access. Expert knowledge normalizes by translating inequality into legitimate difference (performance, score, accreditation).

Mills (1956/2005) shows that knowledge also functions as a device of concentration: elites control not only material resources but also circuits of agenda-setting and diagnosis, articulating government, corporations, and strategic apparatuses. In late modernity, this mechanism intensifies through elite universities, think tanks, consultancies, and specialized media that transform knowledge into agenda power, defining which problems exist and which are “solvable” within technocratic frameworks. Expert knowledge thus not only legitimizes and normalizes but also organizes decision-making by establishing what is “rational” to do and what is “impossible” or “irresponsible.”

Gramsci (1975) adds the mechanism of consent: power is sustained not only by coercion but by hegemony, that is, by the capacity to transform a particular worldview into shared common sense. Knowledge operates as cultural mediation: schools, media, and pedagogical devices institute narratives that render a social order “natural.” This is expressed in global languages such as modernization, innovation, competitiveness, or entrepreneurship, which not only describe but orient subjectivities and expectations, reducing historical alternatives to irrational or unviable deviations.

In this vein, expertise is not merely a repertoire of ideas but a form of institutionalized authority. As Berger and Luckmann (1995) argue, social reality is constructed through processes of institutionalization and legitimation; in late modernity, these processes increasingly rely on expert knowledges that define standards and produce “manageable realities.” Knowledge thus becomes a stratified field of dispute: it can enable critique and emancipation, but it also functions as a mechanism of control by converting historical inequalities into technical parameters.

In global and complex societies, expert knowledge operates as a central resource in the contemporary articulation of power. A sociological mechanism is understood here as a recurrent sequence of institutional operations through which a cognitive resource -in this case, expert knowledge- is translated into observable effects of authority, social classification, and conduct regulation in collective life. These mechanisms do not constitute independent dimensions of power but differentiable processes that link knowledge, institutions, and governmental effects at various levels of social organization.

In a context where access to knowledge is deeply stratified, it becomes a field of social dispute. On the one hand, it can function as an instrument of emancipation, fostering critique and transformation of established structures. On the other, it can operate as a mechanism of control and domination, legitimizing inequalities and naturalizing specific configurations of power. From this perspective, knowledge does not merely describe social reality: it actively intervenes in its structuring by producing criteria of truth, establishing standards of conduct, and enabling technologies of collective management.

**Figure 1:**
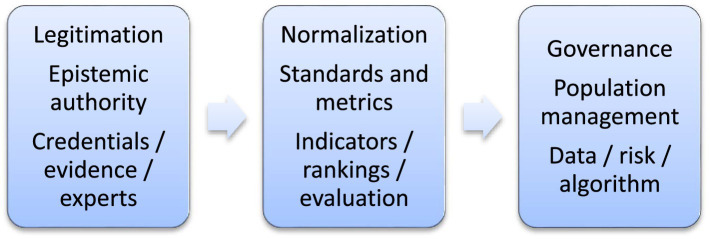
Analytical model of expert knowledge as an articulator of power. Source: author’s own elaboration.

Figure 1 distinguishes three mechanisms that make it possible to understand how expert knowledge articulates power in late modernity: legitimation, normalization, and governance. These processes constitute interdependent sequences that link knowledge, institutions, and governmental effects at different levels of collective life.

Legitimation is understood as the process through which expert knowledge produces epistemic authority and transforms situated potentially contestable- decisions into decisions presented as necessary or inevitable. This mechanism operates at the epistemic–institutional level and materializes in professional credentials, technical protocols, specialized reports, and languages of scientific neutrality. Clear examples can be observed in contemporary monetary policy, where central banks justify interest rate increases as technical responses derived from econometric models, displacing political debate into a technocratic domain.

However, as Bourdieu (1986/2001) warns, legitimacy cannot be reduced to the possession of credentials, since knowledge functions as cultural and symbolic capital that classifies and distributes legitimacy. Collins (1985, 2010) demonstrated that much of scientific production depends on tacit knowledge, while Collins and Evans (2007) distinguished degrees of expertise that explain how certain actors acquire authority in technical debates. Collins (1979/2019), from American sociology, complements this view by showing how the “credential society” reproduces educational inequalities and legitimizes social hierarchies. Legitimation, therefore, is a social process that converts cognitive competencies into recognized authority, naturalizing inequalities under the guise of technical neutrality (Table 1).

**Table 1:** Corpus layers and selection criteria in the genealogical analysis of knowledge-power.

Corpus layer	Function in the analysis	Core texts	Selection criteria
1. Foundational classical core	Establish the seminal categories of power, domination, and legitimacy.	Weber (1922/2021). *Economy and Society*. Gramsci (1975). *Prison Notebooks*. Foucault (1975/1995). *Discipline and Punish*; Foucault (1976/1990). *The History of Sexuality I.*	Texts that inaugurate the paradigms of legal-rational domination, hegemony, and power-knowledge, respectively. They constitute the basis from which the problematic emerges.
2. Development and reconfiguration	Incorporate theories that specify the social mechanisms of knowledge as capital and analyses of governmentality.	Bourdieu (1986/2001). “The Forms of Capital.” Foucault (1978). *Security, Territory, Population*. Rose (1999). *Powers of freedom.*	Works that shift the focus from abstract domination to the practical operation of knowledge as a resource (capital) and as a technology of government (governmentality).
3. Contemporary sociology of knowledge and expertise	Analyze recent transformations linked to quantification, auditing, and technology.	Porter (1995/2020). *Trust in Numbers*. Power (1997). *The Audit Society*. Collins and Evans (2007). *Rethinking Expertise*. Bowker and Star (1999). *Sorting things out.*	Texts that problematize the authority of numbers, evaluation practices, and the social nature of expertise, enabling the connection of theory with phenomena of late modernity.

Source: author’s elaboration.

Normalization corresponds to the process through which expert knowledge establishes standards, metrics, and thresholds that delineate what is acceptable and what is deviant. It translates into indicators, rankings, and evaluation systems that organize institutional and individual conduct. An illustrative example is standardized testing in education, whose results become performance indicators that redefine pedagogical practices and generate dynamics of self-evaluation.

Here, what Bourdieu (1986/2001) calls symbolic violence becomes evident: categories of merit and excellence naturalize inequalities by presenting them as legitimate differences Power (1997, 2004) showed how the proliferation of audits redefines institutional action, while Boltanski and Thévenot (1991/2006) analyzed how actors legitimize their positions through regimes of justification that appeal to criteria of objectivity or efficiency. Normalization, therefore, does not merely measure; it produces differentiated subjects and possible trajectories, consolidating hierarchies under the guise of technical evaluation.

Governance refers to the process through which the infrastructures of knowledge produced by normalization are integrated into technologies of government oriented toward population management. It is articulated with risk management devices, information systems, and algorithms that structure the field of possible decisions. For example, social policy management systems classify populations according to vulnerability and allocate benefits through integrated databases.

Mills (1956/2005) had already warned that elites concentrate organizational and cognitive resources, transforming knowledge into agenda-setting capacity Jasanoff (2004, 2005, 2017) showed how scientific knowledge is articulated with politics and regulation, producing regimes of truth and legitimacy. Didier Fassin (2018) analyzed how public policies generate forms of governance over life and vulnerability. Governance, therefore, is not limited to administering populations: it organizes state intervention through technical categories that define which problems are visible and which solutions are considered rational.

The three mechanisms -legitimation, normalization, and governance are evident in concrete examples and sustained by diverse sociological traditions. Legitimation converts credentials and tacit knowledge into recognized authority; normalization translates that authority into metrics that naturalize inequalities; and governance employs these infrastructures to administer populations through management technologies. In late modernity, marked by expertocracy and datafication, understanding these mechanisms is crucial for analyzing how expert knowledge becomes a central infrastructure of power.

## 4 Knowledge is power: how does knowledge operate to articulate power?

The tradition of critical theory, particularly the Frankfurt School, established a fundamental premise for understanding the relationship between knowledge and power: knowledge does not constitute a neutral reflection of social reality, but rather a field permeated by relations of domination. By linking research and praxis, critical theory argued that modern rationality could become a principle of subjugation when subordinated to the instrumental logic of administration, the market, or political authority (León, 2003). From this perspective, knowledge does not merely describe the social world; it actively participates in legitimating specific forms of power by presenting them as rational, inevitable, or technically necessary.

In this vein, Rusche and Kirchheimer (1939/1984), in Punishment and Social Structure, offer a historical-sociological interpretation of punishment that connects punitive forms with transformations in the economic and social order. Their contribution lies in showing that the penal system cannot be understood as an abstract moral response, but as a historically situated mechanism of social regulation linked to the organization of labor, poverty, and political order. However, this approach presents a relevant limitation for the problem addressed in the present study: by privileging macroeconomic and structural determinants, it tends to undertheorize the everyday mechanisms through which power is exercised within social practices and subjectivities. It is precisely at this level that expert knowledge acquires relevance as a technology of governance.

Marco León’s (2003) diagnosis of the centrality of ideology in this tradition helps explain how domination can operate under concealed forms. Nevertheless, for the purposes of this analysis, the problem cannot be reduced to the issue of “false consciousness.” In late modern societies, a significant portion of power is exercised without the need for explicit ideological deception, but rather through devices that organize social reality via seemingly neutral categories: normal and abnormal, competent and incompetent, risky and safe, efficient and inefficient. In this sense, the central issue shifts from ideology as concealment to knowledge as an infrastructure of veridiction and governance—that is, as a set of practices, classifications, and devices that produce what is considered administrable reality.

It is at this point that the Foucauldian renewal acquires decisive relevance. In the intellectual context following the 1960s, particularly in relation to the political transformations associated with May 1968, Michel Foucault reformulated the analysis of power by displacing it from the figure of a sovereign center toward a relational network distributed across practices, institutions, and discourses. For Foucault, power is not limited to repression; it produces subjects, organizes fields of visibility, delimits what can be said, and establishes regimes of truth. Hence his fundamental thesis: power and knowledge do not constitute separate spheres but co-constitutive dimensions; there is no exercise of power without the production of knowledge, nor effective knowledge that does not presuppose relations of power (Foucault, 1976/1990, 1978, 1980, 2001; Martuccelli, 2013).

In Discipline and Punish, Foucault (1975/1995) shows how modern power is transformed: public torture is replaced by more discreet devices of discipline and surveillance. The prison becomes an organizational model replicated in schools, factories, hospitals, and asylums, where control is exercised through observation, examination, record-keeping, and correction. In these spaces, the objective is not merely to repress conduct but to produce useful and predictable subjects. Knowledge thus becomes an operative technology: observation produces information; information generates classification; and classification enables intervention.

This shift has a central consequence for the present study: if power acts through practices and devices, knowledge does not operate solely as discursive justification but as technology. In other words, knowledge articulates power because it defines what counts as reality, what as deviation, and what as legitimate intervention. For this reason, Foucault (1978) argues that power “penetrates bodies,” induces conduct, and produces discourse; its efficacy derives from its positivity, that is, from its capacity to produce subjects and organize normality.

Nevertheless, to fully understand contemporary configurations of power, it is necessary to complement this perspective with contributions from Science and Technology Studies (STS). This tradition has demonstrated that governmental effects do not emerge from knowledge or technology in the abstract, but from complex sociotechnical networks that articulate experts, artifacts, institutions, standards, and publics (Latour, 2005). From this relational perspective, technical artifacts do not function simply as neutral intermediaries but as mediators that actively participate in the configuration of social relations, redefining problems, stabilizing categories, and reorganizing institutional practices.

In this line, Callon (1984) has shown that the stabilization of knowledge and technology depends on processes of sociotechnical translation, through which human and non-human actors are articulated in networks that define identities, interests, and forms of collective action. Within these networks, standards and classification systems play a central role. As Bowker and Star (1999) argue, infrastructures of classification—statistical categories, indicators, protocols, or rankings—do not merely describe social reality; they produce it by establishing frameworks of comparability that organize institutional practices and distribute recognition and exclusion.

A decisive element in this dynamic is the growing centrality of quantification. Porter (1995/2020) has shown that the authority of numbers is linked to their capacity to generate trust in contexts of uncertainty, allowing complex decisions to be presented as objective and comparable. In this sense, the contemporary expansion of indicators, audits, and metrics constitutes an epistemological infrastructure that legitimates political decisions under the language of technical neutrality.

Within this framework, the work of Harry Collins (1985, 2010) is particularly relevant for understanding the social nature of expertise. Collins has demonstrated that expert knowledge is grounded in forms of tacit knowledge that cannot be reduced to explicit rules or formalized information. Expert authority emerges within communities of practice where cognitive competencies are distributed, configuring what Collins and Evans (2007) describe as the social division of cognitive labor. From this perspective, expertise is not a pre-existing attribute of the expert actor, but a relational achievement stabilized through technical devices, institutional practices, and processes of collective recognition.

From this expanded perspective, expert knowledge articulates power through three interdependent mechanisms:

- Legitimation, through the production of epistemic authority that transforms disputable decisions into decisions presented as technically necessary, supported by credentials, evidence, and procedures.
- Normalization, through the establishment of standards, categories, and metrics that enable comparison, classification, and correction of conduct within institutions and populations.
- Governance, through the integration of these metric infrastructures into risk management devices, data systems, and algorithms that orient public and private decisions.

These mechanisms do not operate as separate stages or as a linear sequence. On the contrary, they are co-constituted within sociotechnical assemblages in which experts, institutions, and artifacts jointly participate in the production of authority, standards, and decisions. Legitimation generates trust in expert knowledge; normalization translates that authority into comparative infrastructures; and governance employs those infrastructures to organize collective decisions. In turn, the outcomes of these interventions feed back into the authority of expert knowledge, reinforcing the stability of the system.

Several examples illustrate this simultaneity: in the field of public security, predictive policing algorithms not only govern by anticipation but also redefine what counts as “risk” and legitimate police decisions under the language of technique. In education, rankings and standardized evaluations produce institutional legitimacy, normalize student performance, and govern the actions of schools and universities through incentives and sanctions. In health, clinical protocols and hospital management systems legitimate medical practices, normalize bodies and behaviors, and govern populations through risk and efficiency indicators.

These cases show that the simultaneity of legitimation, normalization, and governance is not a spontaneous effect, but the result of sociotechnical infrastructures of stabilization that articulate experts, institutions, artifacts, and publics. From this perspective, contemporary power–knowledge can be understood as a relational fabric that produces authority, fabricates comparable subjects, and orients collective action in a single movement.

## 5 Between critical theory and genealogical critique

The contemporary articulation between knowledge and power requires a rereading that does not merely add together theoretical traditions, but rather explores their internal tensions and analytical capacity to explain current forms of expert governance. The challenge lies in reconstructing how knowledge ceases to operate solely as “ideology” or “superstructure” and begins to function as a practical infrastructure of social conduct, capable of organizing behaviors, producing subjects, and administering populations. This shift from power understood as domination to power understood as government constitutes precisely the point of convergence between critical theory and genealogical critique.

From the perspective of critical theory, knowledge appears as a field permeated by relations of domination: modern reason, when transformed into instrumental reason, tends to subordinate the social to logics of calculation, administration, and performance, thereby weakening the emancipatory capacity of reflection (Horkheimer and Adorno, 2002; Horkheimer, 1969/2004). This tradition provides a fundamental insight: domination is reproduced when the historical is presented as necessary, when political decisions are translated into technical imperatives, and when critique is neutralized under the languages of efficiency and management. However, when analysis focuses exclusively on ideology or macrostructural determinants, it risks underestimating the everyday productivity of power—how it operates within practices, institutions, and subjectivities, even without recourse to direct coercion.

Genealogical critique, inspired by Nietzsche and developed by Foucault, radicalizes this problem by arguing that knowledge is not simply “at the service” of power, but intrinsic to its exercise. In Nietzsche, the critique of the ideal of truth as correspondence introduces a decisive thesis: forms of knowledge are tied to forces, interests, and struggles to impose valuations; they are neither neutral nor universal, but historical and agonistic (Nietzsche, 1887/1998). Foucault takes up this gesture and transforms it into a research program oriented toward analyzing how regimes of truth are constituted that enable practices of government, producing subjects, normalities, and objects of intervention (Foucault, 1976/1990, 1975/1995, 1964/2015, 2006).

From this perspective, power should not be understood solely as repression, but as a practical rationality that organizes the social world. It produces categories such as “normal/abnormal,” “capable/incapable,” “healthy/risky,” institutes procedures of examination, record, and archive, and stabilizes hierarchies through devices that render individuals legible and governable (Foucault, 1976/1990, 1975/1995, 1964/2015; Weber 1922/2021). Knowledge, therefore, does not simply describe reality: it contributes to constructing it as an administrable field.

On this basis, the contemporary functioning of power can be analyzed through three interrelated operations of expert knowledge:

- Legitimation: the production of epistemic authority that defines what counts as valid evidence and who is authorized to interpret it. Through professional credentials, methodological procedures, and institutional standards, disputable decisions are transformed into decisions presented as technically necessary. Obedience is reformulated as technical competence (Collins and Evans, 2007).
- Normalization: the establishment of thresholds, classifications, and metrics that define what is acceptable and what is deviant. Examination, surveillance, and record-keeping produce comparable subjects susceptible to correction and self-adjustment. Normalization operates not only through prohibitions but also through mechanisms of comparison and evaluation that induce individuals to align with institutional standards (Foucault, 1975/1995; Bowker and Star, 1999).
- Governance: an analytical shift beyond disciplinary normalization. While normalization regulates individual conduct, governance refers to the integration of these metric infrastructures into decision-making systems that orient the management of populations. Indicators, databases, predictive models, and algorithmic systems enable the administration of risks, allocation of resources, and prioritization of interventions on a large scale (Miller and Rose, 2008; Espeland and Stevens, 2008).

This point requires an important conceptual clarification. The concept of governance is not intended to replace or replicate Foucault’s notion of governmentality. Indeed, population management, risk administration, and the optimization of collective life already form part of Foucault’s analysis of biopolitics. However, the argument advanced here is that contemporary forms of expert governance introduce an additional transformation: the conduct of populations is increasingly organized through sociotechnical infrastructures of quantification and datafication that simultaneously reconfigure processes of legitimation and normalization. In this sense, governance does not simply constitute an extension of Foucauldian governmentality, but the space where the other two dimensions are reorganized. Numbers, indicators, and algorithms not only administer populations; they also redefine what counts as legitimate evidence and what is considered normal behavior (Porter, 1995/2020; Power, 1997, 2004).

Studies on “governance by numbers” and “audit societies” show that power is increasingly exercised through metrics that translate political decisions into apparently neutral technical indicators (Power, 2004; Espeland and Stevens, 2008). Likewise, recent analyses of algorithmic power emphasize that systems of automatic classification and statistical prediction do not merely describe social behaviors, but reorganize them by redistributing opportunities, risks, and forms of institutional intervention (Beer, 2017; Couldry and Mejias, 2019). Thus, contemporary governance can be understood as a sociotechnical reconfiguration of power, in which legitimation, normalization, and population management are articulated through infrastructures of data, metrics, and algorithms.

In this way, domination in late modernity does not disappear; it is transformed into a regime of cognitive and technical government. Expert knowledge articulates power because (a) it authorizes decisions, (b) it standardizes conduct, and (c) it administers populations. Its analytical value lies not in identifying three separate dimensions, but in showing how they are co-constituted in feedback circuits sustained by sociotechnical infrastructures of governance. The analysis of power–knowledge must therefore explain how, in contemporary institutions, expertise simultaneously produces authority, normality, and governability, continuously reorganizing what is considered evidence, acceptable conduct, and legitimate objects of intervention.

## 6 Discussion: expert knowledge, contemporary rationalities, and forms of government

The analysis developed here supports the claim that the principal deficit of classical sociology of power does not lie in the absence of critical categories, but in the analytical fragmentation with which the relationship between knowledge and power has historically been addressed. While some traditions privileged domination and ideology, and others emphasized discipline or subjectivation, late modernity requires a framework capable of explaining the simultaneous, articulated, and persistent operation of knowledge as legitimation, normalization, and governance. It is precisely at this point that dialogue with contemporary sociology becomes decisive for evaluating the explanatory scope of the proposal.

Studies of governmentality, developed primarily by Nikolas Rose and collaborators, have shown that power in advanced societies operates less and less through direct orders and increasingly through indirect forms of conduct, supported by expert knowledges that orient the actions of individuals, institutions, and collectives (Rose, 1999; Miller and Rose, 2008). From this perspective, knowledge does not merely justify political decisions ex post, but structures the fields of possible intervention, delimiting which problems exist, which solutions are thinkable, and which behaviors are considered rational. The notion of governmentality allows us to understand how knowledge acts as a technology of government, though it tends to describe a diffuse functioning without systematically integrating its different operations. The contribution of the present work lies precisely in organizing this dispersed functioning into three analytically distinguishable mechanisms, without losing sight of their empirical interdependence.

Wendy Brown’s critique of neoliberal rationality reinforces this diagnosis. For Brown (2015), neoliberalism should not be understood solely as an economic program, but as a normative rationality that reconfigures the subject, the state, and democracy through languages of efficiency, competition, and human capital. Within this framework, expert knowledge fulfills a dual central function: it legitimates political decisions by presenting them as inevitable technical imperatives, and it normalizes entrepreneurial, self-responsible, and competitive subjectivities, displacing democratic deliberation into the terrain of management. Neoliberal rationality, as analyzed by Brown, clearly demonstrates that knowledge no longer operates merely as ideology, but as an organizing principle of social life, confirming the need to explicitly conceptualize its function of governance.

From a biopolitical perspective, Thomas Lemke (2011) deepens this analysis by showing that contemporary government is increasingly exercised through the management of life, risk, and population, articulating biomedical, economic, and statistical knowledges. Biopolitics does not replace discipline but reconfigures it: it is no longer only about correcting deviations, but about optimizing behaviors, anticipating threats, and administering probabilities. In this context, expert knowledge becomes an infrastructure of preventive intervention, where indicators, diagnoses, and predictive models define political priorities, resource allocations, and social hierarchies. The argument advanced here is that this biopolitical rationality cannot be fully understood without integrating its dimensions of legitimation (expert authority), normalization (risk standards), and governance (population management).

A complementary line is the critique of psychopolitics developed by Byung-Chul Han. According to Han (2014, 2018), contemporary power no longer operates primarily through prohibition or repression, but through self-exploitation, seduction, and the production of desire. In this scheme, knowledge-especially psychological, behavioral, and algorithmic- aims to optimize subjective performance, turning freedom into a resource of control. Although his approach has been questioned for its normative tone, its contribution is significant, as it highlights how expert knowledge produces subjects who govern themselves according to performance metrics, reinforcing the thesis that contemporary domination is increasingly exercised as internalized normalization rather than external imposition.

Recent debates on algorithmic power and datafication deepen this problem further. Research by Beer (2017) and Couldry and Mejias (2019) has demonstrated that algorithms are not neutral tools, but operationalized forms of knowledge that classify, prioritize, and exclude. The automation of decisions in public policy, labor markets, credit systems, security, or health constitutes an emerging modality of governance based on data, where knowledge materializes in technical infrastructures that redistribute opportunities and risks without direct democratic mediation. This phenomenon highlights, with particular clarity, the conceptual gap that this work seeks to address: no theory focused exclusively on coercion, ideology, or class is sufficient to explain these dynamics.

Convergently, the sociology of quantification and auditing has shown that numbers function as devices of power. Power (1997) and Espeland and Stevens (2008) demonstrate that indicators, rankings, and evaluations do not merely measure reality, but produce it, orienting institutional and subjective conduct. In these cases, expert knowledge operates simultaneously as legitimation (“what is not measured does not exist”), as normalization (permanent comparison), and as governance (allocation of resources, incentives, and sanctions). These empirical findings reinforce the central thesis of the present work: knowledge has become an integral technology of government.

Finally, from the sociology of knowledge and science, Sheila Jasanoff (2011, 2017) and Saskia Sassen (2014) have emphasized that global regimes of knowledge configure new forms of authority and inequality. Expertocracy is not distributed homogeneously, but concentrated in certain territories, institutions, and transnational networks, reproducing asymmetries between centers and peripheries of knowledge. This global dimension confirms that knowledge not only organizes everyday life but also structures hierarchies on a planetary scale, reinforcing the relevance of a conceptual rearticulation that can account for its political function in contexts of global interdependence.

Expert knowledge functions simultaneously as a source of legitimacy, a matrix of normalization, and a technology of governance because it has become a practical rationality that organizes social action, defines standards of conduct, and administers populations through technically and culturally legitimized devices. The contribution of this work therefore lies in integrating these operations into a single analytical framework, capable of explaining contemporary phenomena such as governance by indicators, technocratic risk management, datafication, and decision automation-without reducing power to coercion, ideology, or class, but also without denying their historical relevance.

## 7 Conclusion

The present study aimed to conceptually rearticulate the nexus between knowledge and power in order to understand its functioning in late modern societies, characterized by the expansion of expertocracy, neoliberal rationality, and the growing mediation of sociotechnical infrastructures based on data and algorithms. The theoretical trajectory developed here supports the claim that transformations associated with cognitive capitalism and the datafication of social life have substantively altered the ways in which power is legitimized, exercised, and reproduced. In this context, the classical categories of the sociology of power—centered on coercion, ideology, or class structure—prove insufficient to explain how authority, obedience, and social regulation are currently stabilized through expert knowledges and technical devices operating under the language of neutrality, evidence, and efficiency.

The critical dialogue between the sociocritical tradition and Foucauldian genealogy made it possible to identify both analytical convergences and explanatory limits in classical approaches to the knowledge–power relation. Critical theory demonstrated that modern rationality can become an instrument of domination when historical decisions are presented as inevitable technical imperatives, while Foucauldian genealogy revealed that power does not operate solely through prohibitions or external coercion, but through practices, devices, and regimes of truth that produce subjects, normalities, and fields of intervention. Yet, taken separately, both traditions leave open the question of how expert knowledge currently articulates forms of governance in contexts marked by massive quantification, datafication, and decision automation.

To address this conceptual gap, the article proposed an analytical rearticulation of the knowledge–power nexus through the identification of three interdependent mechanisms: legitimation, normalization, and governance. Legitimation refers to the production of epistemic authority that transforms disputable decisions into decisions presented as technically necessary; normalization describes the establishment of standards, metrics, and thresholds that produce comparable and evaluable subjects; and governance denotes the integration of these infrastructures of knowledge into population management devices based on indicators, data, and predictive models. The analytical relevance of this distinction lies not in separating dimensions of power, but in showing how these operations are articulated in feedback circuits sustained by sociotechnical networks linking experts, institutions, artifacts, and publics.

From this perspective, one of the central conclusions of the study is that expert knowledge does not merely accompany power, but constitutes one of its conditions of possibility. In late modernity, power is increasingly exercised through institutionalized knowledges that produce epistemic authority, define criteria of truth, and delimit legitimate fields of intervention. The old adage “knowledge is power” thus acquires a more precise meaning: knowledge not only grants power, but constitutes a specific form of its exercise, materialized in norms, standards, technical devices, and socially validated institutional practices.

The analysis further demonstrated that contemporary domination operates simultaneously as legitimation, normalization, and governance. Political decisions are transformed into technical imperatives through processes of epistemic legitimation; social practices are reorganized through standards and metrics that produce comparable subjects; and populations are administered through data infrastructures, classification systems, and risk management devices. These operations do not unfold linearly, but mutually reinforce one another in sociotechnical circuits that articulate experts, institutions, and knowledge technologies.

In this context, recent developments associated with quantification, permanent auditing, datafication, and the expansion of decision algorithms do not represent a rupture with classical forms of power, but rather a technical reconfiguration of them. Numbers, indicators, and algorithmic systems constitute forms of operationalized knowledge that redistribute opportunities, hierarchies, and risks, while reducing the space of political deliberation by translating normative conflicts into apparently technical problems. In this way, power becomes less visible but potentially more effective, inscribed in infrastructures that enable governance at a distance through devices of evaluation, prediction, and permanent comparison.

Expert knowledge produces governable subjectivities through processes of internalization of the norm. Performance metrics, evaluation systems, and devices of permanent comparison induce individuals to self-observe, self-evaluate, and self-regulate according to criteria of efficiency, performance, and individual responsibility. In this sense, contemporary domination can largely dispense with direct coercion, as power is exercised through forms of internalized normalization that transform obedience into self-regulation.

Taken together, these findings suggest that understanding power in contemporary societies requires advancing toward analytical frameworks capable of capturing the complexity of knowledge-based governance. The conceptual proposal developed here—articulated around the mechanisms of legitimation, normalization, and governance—constitutes a contribution in this direction, integrating insights from critical theory, Foucauldian genealogy, and contemporary sociology of knowledge. Rather than closing the debate, this framework seeks to open a research program oriented toward empirically analyzing how expert knowledge organizes institutions, cultures, and everyday lives in contexts of inequality, globalization, and technological mediation.

Ultimately, the contribution of this work to the field of the sociology of power and knowledge lies in shifting the classical question of who holds power toward the analysis of how power is exercised through knowledge. Examining expert devices, technical rationalities, and cognitive infrastructures that structure contemporary social life allows not only for a more precise understanding of current forms of domination, but also for opening a critical space to interrogate the possibilities of democratizing knowledge, fostering social resistance, and collectively reappropriating processes of governance.

## Data Availability

The original contributions presented in the study are included in the article/supplementary material, further inquiries can be directed to the corresponding author.
